# Comparative Genome Sequence Analysis of *Actinobacillus pleuropneumoniae* Serovar 8 Isolates From Norway, Denmark, and the United Kingdom Indicates Distinct Phylogenetic Lineages and Differences in Distribution of Antimicrobial Resistance Genes

**DOI:** 10.3389/fmicb.2021.729637

**Published:** 2021-09-10

**Authors:** Liza Miriam Cohen, Janine T. Bossé, Marc Stegger, Yanwen Li, Paul R. Langford, Camilla Kielland, Thea Blystad Klem, Stine Margrethe Gulliksen, Birgit Ranheim, Carl Andreas Grøntvedt, Øystein Angen

**Affiliations:** ^1^Department of Production Animal Clinical Sciences, Faculty of Veterinary Medicine, Norwegian University of Life Sciences (NMBU), Oslo, Norway; ^2^Section of Paediatric Infectious Disease, Department of Infectious Disease, Imperial College London, London, United Kingdom; ^3^Department of Bacteria, Parasites and Fungi, Statens Serum Institut, Copenhagen, Denmark; ^4^Norwegian Veterinary Institute, Oslo, Norway; ^5^Animalia, Oslo, Norway

**Keywords:** *Actinobacillus pleuropneumoniae*, phylogeny, whole genome sequencing, antimicrobial resistance genes, evolution, serovar 8

## Abstract

*Actinobacillus pleuropneumoniae* is the etiological agent of porcine pleuropneumonia, a disease of major impact on pig health, welfare, and productivity globally. Serovar 8 (APP) is the predominant clinical serovar in Norway and the United Kingdom (UK), and has been isolated from clinical cases in Denmark. The primary objective of this study was to characterize the genetic variability of isolates of *A. pleuropneumoniae* APP8 in the Norwegian population. The secondary objectives were to determine the within-host variability of APP8; to compare the APP8 bacterial populations in Norway, Denmark, and the UK, including antimicrobial resistance (AMR) gene profiles and to assess the effect of national differences in antimicrobial drug use and restricted animal movement on the occurrence of resistance. Isolates of APP8 from the UK (*n*=67), Denmark (*n*=22), and Norway (*n*=123) collected between 1983 and 2020 were compared using whole genome sequencing. To investigate genetic variability within individual hosts, an additional 104 APP8 isolates from the lungs of six Norwegian pigs were compared. Very low within-host variation was observed (≤ 2 single nucleotide polymorphisms). The phylogeny of 123 Norwegian APP8 isolates from 76 herds revealed some within-herd genetic variation, but substantial geographical clustering. When inferring the relatedness of the three international APP8 collections, the topology highlighted the existence of two distinct monophyletic branches characterized by the Norwegian and UK isolates, respectively. Three Danish isolates were scattered across the UK branch, whereas the remaining 19 Danish isolates clustered in two monophyletic groups nested in the Norwegian branch. Coalescence analysis, performed to estimate the divergences from a common ancestor, indicated a last common ancestor several centuries ago. The phylogenetic analyses also revealed striking differences in occurrence of AMR genes, as these were 23-times more prevalent among the UK isolates than among the Norwegian isolates. An increased understanding of the effects of population strategies is helpful in surveillance and control of infectious diseases.

## Introduction

Comparing genome sequence data provides information on molecular and epidemiologic relationships. In microbial infection dynamic studies, population genomics is used to gain insight into species populations, to map diversity and better understand the transmission patterns of pathogens. Genetic variability can be compared at many levels within and between species populations and is influenced both by inherent biologic characteristics that affect the transmission of the pathogen, as well as by host population structures and events (i.e., host population dynamics). Transmission routes, animal population structures and management practices, and patterns of animal movements should all be reflected in the genetic relationships observed between the pathogens.

Systemizing the pig production sector enables efficient surveillance and biosecurity measures which are becoming increasingly important. In order to secure a high level of biosecurity, strict population structures are applied to the pig production in many countries (including Norway, the UK, and Denmark), which include restricted or negligible live animal import from other countries and domestic trade structured through a tiered pyramid. The Norwegian pig production system has a pyramidal structure, with a unidirectional flow of animals from a low number of genetic nucleus breeding herds at the top, to a larger number of commercial producers at the bottom (Norwegian Veterinary Institute, 2021a). *Actinobacillus pleuropneumoniae* is a porcine opportunistic bacterium and the etiological agent of porcine pleuropneumonia. *A. pleuropneumoniae* is transmitted mainly through direct contact between animals, commonly from sows to suckling piglets, or by aerosols over short distances (Chiers et al., 2002; Velthuis et al., 2003; Fablet et al., 2011; Tobias et al., 2014). In most modern pig producing countries, herds are endemically infected with *A. pleuropneumoniae,* with healthy carrier pigs harboring the bacterium in their tonsils (Gottschalk, 2015; Sassu et al., 2017). While more virulent isolates are able to colonize the lower respiratory tract and cause pleuritis and/or pneumonia, other isolates will not (Gottschalk, 2015). To what extent bacteria involved in lung infections are genetically heterogeneous or are solely monoclonal has not been elucidated.

Porcine pleuropneumonia is considered a major health and welfare challenge to pig production worldwide and is a source of considerable use of antimicrobial drugs, both in treatment and prophylaxis (Sassu et al., 2017). As prevalent use of antimicrobial drugs to combat disease leads to emergence of resistant strains (FAO, 2016), national strategies for antimicrobial drug use are based on knowledge of the antimicrobial resistance (AMR) profiles of relevant pathogens. Antimicrobial treatment practices are an important population-wide factor affecting genetic variability through selection of resistance in pathogenic, as well as commensal, bacteria. Population-wide surveillance for AMR genes using whole genome sequencing (WGS) can be useful when forming future national strategies for treatment and control. WGS is a sensitive method for detecting known AMR genes in bacteria (Anjum et al., 2017) including *A. pleuropneumoniae*, where the AMR genotype was shown to correlate nearly 100% with the phenotype for antimicrobial agents other than macrolides (Bossé et al., 2017).

Among the 19 described serovars of *A. pleuropneumoniae* (Stringer et al., 2021), serovar 8 (APP8) is most commonly isolated from cases of acute porcine pleuropneumonia in Norway (Cohen et al., 2020; Norwegian Veterinary Institute, 2021b) and the UK (O’Neill et al., 2010; Li et al., 2016). In Denmark, APP8 has also been isolated from clinical cases, although earlier studies have shown it is not the most prevalent serovar (Møller et al., 1992; Kokotovic and Angen, 2007). Previous analyses of *A. pleuropneumoniae* populations, using multilocus enzyme electrophoresis (Møller et al., 1992; Hampson et al., 1993) or amplified fragment length polymorphism (Kokotovic and Angen, 2007), indicated that the species is divided into clonal groups mainly corresponding to the different serovars. More recent analysis by enterobacterial repetitive intergenic consensus-based PCR, revealed a degree of variation within populations of isolates of serovars 1, 7, and 15 in Australia (Yee et al., 2018). Comparison of whole genome sequences from seven Brazilian APP8 isolates indicated that differences in these were mainly due to prophage and other mobile genetic elements (Prado et al., 2020). To our knowledge, there has previously not been published detailed analysis of large populations of a single serovar of *A. pleuropneumoniae* using genomic data.

The primary objective of this study was to characterize the genetic variability in isolates of APP8 in the Norwegian population. Secondary objectives included determining variability at two further levels, i.e., within-host and between populations in different countries (Norway, Denmark, and the UK), and comparing AMR genes in the different national populations as an indicator of the effect of regional antimicrobial drug use and closed populations on dissemination of AMR. We used temporal and geographic data to gain knowledge regarding the effect of the pig population structure, animal movement, antimicrobial drug consumption and AMR levels on the evolution of this important pig pathogen.

## Materials and Methods

### Bacterial Isolates

Isolates of APP8 from countries where this serovar has commonly occurred in clinical cases were included in this study. A total of 316 isolates were included, of which 227 isolates originated from Norway, 67 originated from the UK and 22 from Denmark (Table 1).

**Table 1:** APP8 sample population grouped according to country of origin.

Country of origin	Isolate repository for phylogenetic reconstruction^*^	Additional isolates sampled for within-host analysis	Total
Norway	123	104	227
UK	67	0	67
Denmark	22	0	22
Total	212	104	316

* Isolates stem from routine diagnostics.

#### The Norwegian Isolate Repository

A primary isolate repository was established, consisting of 123 APP8 isolates from individual pigs collected through routine diagnostics at the Norwegian Veterinary Institute (NVI) in the period from 2004 to 2019. The isolates originated from a total of 76 herds, of which 23 had given rise to multiple (range 2–6) isolates. These isolates mainly stemmed from cases of clinical pleuropneumonia; however, a minority of isolates (*n*=2) were cultured by swabbing from pneumonic lungs at slaughter with no prior remarks of clinical signs. Serovar determination was performed by the method as previously described (Cohen et al., 2020). Non-APP8 isolates (*n*=5), collected at the NVI in the same period, were excluded from the study. In 2019 and 2020, an additional 104 isolates were sampled from pneumonic lungs at the NVI to investigate within-host variation of *A. pleuropneumoniae*. We sampled six pigs from five geographically unrelated herds by swabbing two to five lesions within every set of lungs. Swabs from each lesion were cultured on individual agar plates. From each plate we selected five to eight colonies of *A. pleuropneumoniae*, all serovar 8, resulting in 104 isolates all of which were sequenced.

Swabbed material from lungs and pleura was cultured on 5% sheep’s blood on agar base including a cross-streak of β-toxic *Staphylococcus aureus* to support the growth of nicotinamide adenine dinucleotide-dependent *A. pleuropneumoniae* and incubated in a humidity chamber in 5% CO_2_. Colonies were purified by secondary culturing, then stored at −80°C. Isolates were revived by the same bacteriological procedures. Colony identification was verified by matrix assisted laser desorption ionization (MALDI) time-of-flight mass spectrometry (MALDI Biotyper, Bruker Daltonics, Bremen, Germany). Pure cultures were collected onto sterile swabs and placed in Amies transport medium with charcoal and shipped in bulk by courier to Statens Serum Institut (SSI), Denmark. Prior to DNA extraction, isolates were cultured on chocolate agar and incubated at 35°C over-night.

#### The UK Isolate Repository

Genomic sequences from 67 clinical isolates of APP8 have been included from the archives of Imperial College in London. The genome for the APP8 reference strain 405, GenBank accession ID: txid754257, was included here as a UK isolate, although it was isolated in Ireland in 1984 (Nielsen and O’Connor, 1984). Retrieval of the UK isolates was performed from 2003 to 2011 at the Animal and Plant Health Agency (formerly known as the Animal Health and Veterinary Laboratories Agency) diagnostic laboratories in England and Wales. Methods regarding sample collection and handling of the UK isolates have been published previously (Bossé et al., 2015).

#### The Danish Isolate Repository

Twenty-two Danish clinical isolates of APP8 originate from diagnostic work at Danish Veterinary Laboratory (later National Veterinary Institute – Technical University of Denmark), over the years from 1983 to 2009. Eight of these isolates (sampled between 1983 and 1991) were shipped to Imperial College, London, United Kingdom, in 2007. DNA extraction and WGS of these isolates were performed according to the method previously described (Bossé et al., 2020). The remaining 14 isolates (sampled between 1996 and 2009) were transferred to SSI in 2019 and cultured on chocolate agar, and incubated at 35°C over-night, prior to DNA extraction.

### Metadata

The year of sampling was retrieved from the diagnostic records for all included isolates (*n*=316).

#### Epidemiology of the Norwegian Isolates

We retrieved the unique farm identification number (ID) for the farm from which the Norwegian APP8 pig isolates (*n*=123) originated. This ID was in turn used to identify the farm location, production type (herd category) and abattoir affiliation as livestock movement is restricted within the slaughterhouse systems. This information was included to study the effects of geographic origin and livestock trade on genetic variability on population level.

The variable farm location was divided into five geographical categories: North (*n*=6), Central (*n*=21), East (*n*=37), South-West (*n*=46), and Greater Oslo (*n*=12). For one isolate, this information was unavailable. The geographic regions defined here are based on the official administrative regions of the Norwegian Food Safety Authorities, as they were in April of 2020 (Norwegian Food Safety Authorities, 2012). For statistical analyses, these groups were merged to form three geographical regions (Supplementary Figure 1) with larger sample sizes: East and Greater Oslo (Region 1), South-West (Region 2), North and Central (Region 3).

Herd of origin was categorized into four types (Table 2), based on the structure of the Norwegian pig production pyramid system. The category “Breeding herds” included isolates from genetic nucleus and multiplier breeding herds (*n*=8), while the “Commercial herds” category included isolates from commercial sow herds and fattening pig herds for consumption (*n*=101). A 3rd category labeled “Other” (*n*=8), included isolates from places of origin that differed from the common herd types in the production pyramid, such as isolates from stud quarantine and testing stations, as well as pigs submitted for diagnostics by an abattoir and could not be traced to a herd. Six isolates were grouped as “Unknown.”

**Table 2:** An overview of the distribution of Norwegian APP8 isolates (*n*=123) divided into two categories; the abattoir and herd of origin.

Abattoir category	No of herds (%)	No of isolates (%)	Number of isolates in herd of origin (% of total)			
			Breeding herds	Commercial herds	Other	Unknown
Private	28 (36.8)	42 (34.1)	2 (1.6)	39 (31.7)	1 (0.8)	0
Cooperative	46 (60.5)	78 (63.4)	6 (4.9)	61 (49.6)	7 (5.7)	4 (3.3)
Unknown	2 (2.6)	3 (2.4)	0	1 (0.8)	0	2 (1.6)
Total	76	123	8 (6.5)	101 (82.1)	8 (6.5)	6 (4.9)

The variable for abattoir affiliation was divided in three categories: “Cooperative,” owned by the members (Nortura, *n*=78), “Private” (privately owned abattoirs, members of The Meat and Poultry Industry’s National Association, *n*=42), or “Unknown” (*n*=2; Table 2). These categories were chosen because livestock trade in Norway is usually restricted within these abattoir systems.

Summary data for the tables and statistical analyses of the epidemiological data were performed using Stata (STATA SE/15 for Windows; Stata Corp., College Station, TX, United States). The distribution of geographic regions of origin and abattoir affiliation within three clades in the Norwegian APP8 phylogeny was assessed using a cross table and evaluated using the chi squared (χ^2^) test. One isolate was excluded from the analysis, as the region of origin was unknown. Herd as a random effect was accounted for.

A dataset of the isolate metadata has been included (Supplementary Data).

### Analyses of Genetic Variability

Bioinformatic analysis of bacterial genome sequences allow study of genetic variability within and between populations. In this study we assessed genetic variability in whole genome sequences through single nucleotide polymorphisms (SNPs) in the core genome of the included isolates. WGS was also used to characterize the AMR genes carried by the isolates.

#### Genome Sequencing

Genomic DNA was extracted from the Norwegian (*n*=227) and 14 of the Danish isolates using the DNeasy Blood and Tissue Kit (QIAGEN, Germantown, MD, United States) and quantified on a Qubit 3.0 Fluorometer (Invitrogen, Waltham, MA, United States). Preparation of the DNA sequence libraries were performed using the Illumina Nextera XT DNA Library Preparation Kit (Illumina Inc., San Diego, CA, United States) and sequenced on a NextSeq 500 platform (Illumina Inc., San Diego, CA, United States) with paired-end sequencing (2×151bp) using a 300-cycle NextSeq Mid-Output Kit followed by quality assessment using bifrost.^1^ The reads were *de novo* assembled using SPAdes (Bankevich et al., 2012) using default parameters.

#### Phylogenetic Reconstruction Using SNP Calling

For phylogenetic reconstruction, the sequence reads from all isolates were included. Using the closed chromosomal sequence of APP8 strain MIDG2331 (GenBank accession number LN908249.1) as reference, after removal of duplicated regions using NUCmer (Kurtz et al., 2004), identification of SNPs was performed with NASP v.1.0.0 (Sahl et al., 2016). All positions with less than 10-fold sequencing depth and 90% unambiguous variant calls for any isolate were excluded. Removal of high-density SNP regions, such as those caused by recombination, were identified and removed using Gubbins v.2.3.4 (Croucher et al., 2015) prior to phylogenetic reconstruction using IQ-TREE v.1.5.5 (Nguyen et al., 2015) using ModelFinder as implemented in IQ-TREE, and phylogenetic robustness was assessed with bootstrap analysis using 100 replicates. Visualization and annotation of the phylogenies were performed using iTol v4.314.^2^ Geographic visualization was performed in Microreact.^3^ In Microreact, each isolate was given the coordinates for the capital of the municipality in which the farm was located to comply with General Data Protection Regulations. Additionally, a SNP distance between multiple isolates collected from the same pigs was obtained as outlined above and used to investigate within-host variation, using the same-sized core genome. Mean SNP counts for within-pig (internal) variability were calculated (Table 3). Isolates were considered clonal if they displayed very limited variation (≤2SNPs).

**Table 3:** Internal SNP differences among 104 APP8 isolates from six pigs from five different herds.

Pig	No isolates	Internal SNP distance
1	25	0
2^*^	10	0-1
3^*^	10	0
4	14	0
5	20	0–2
6	25	0–2

* Clonal isolates from the same herd, sampled during a disease outbreak.

### Genotyping AMR

The presence of AMR genes was investigated using ABRicate^4^ to search the assembled genomes for genes associated with resistance found in the ResFinder database (Zankari et al., 2012). The gene presences were determined based on a combined >80% hit length and >90% sequence identity. Only the primary repository APP8 isolates (*n*=212) were included in these analyses (Table 1).

### Coalescence Analyses

To ascertain the temporal relationships of our samples, we utilized coalescent analyses that model how variants sampled from a population may have originated from a common ancestor, by estimating rooted, time-measured phylogenies. For this, BEAST v2.6 was used and run at the CIPRES Science Gateway v3.3^5^ public resource. The bModelTest v1.2.1 package was applied using the transitionTransversionSplit model and automatic estimation of mutation rate and log normal nucleotide frequencies. Different coalescent (constant population and Bayesian skyline) and clock models (strict clock and relaxed clock log normal) were applied to allow both constant and variable mutation rates across the branches. For all combinations of models, independent chains (*n*=2) of 400million in length were performed with storing of data every 40,000th step, assessing the convergence of key ESS values using Tracer v1.7, after burn-in of 10% by calculation of the log_10_ Bayes factors for model comparison. Trees were visualized using DensiTree v2.2.7 (Bouckaert, 2010).

## Results

### Within-Population Variation of Norwegian APP8 Isolates

#### Phylogeny

**Figure 1:**
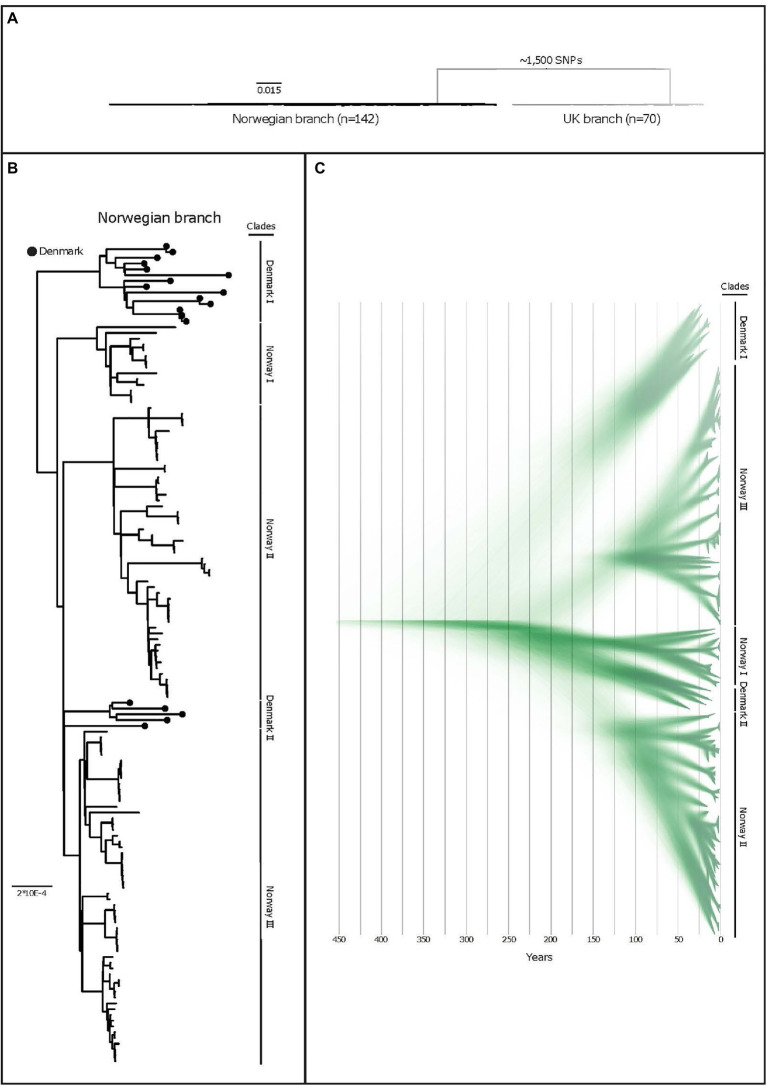
**(A)** A purged midpoint rooted maximum likelihood phylogeny of isolates of APP8 from Norway, illustrating the two distinct branches of the APP8 collection (the Norwegian branch and the UK branch). The Norwegian branch contains 19 Danish and 123 Norwegian isolates (*n*=142), whereas the UK branch contains three Danish and 67 UK isolates (*n*=70) with ~1,500 SNPs separating the branches. **(B)** A rooted maximum-likelihood phylogeny of the Norwegian APP8 branch. Danish isolates are indicated by black dots on the branches. Based on the topology, five distinct phylogenetic clades labelled Norway I-III and Denmark I and II are highlighted. The scale indicates substitutions per site. **(C)** DensiTree representation of 9,000 time-measured phylogenies obtained from BEAST with the best fitting model after removal of 10% burn-in. X-axis is a time scale indicating years since 2019, illustrating the temporal relationships between the 123 Norwegian and 19 Danish isolates. Similar clustering is observed as with the maximum-likelihood approach, however in a different order, highlighted by clade labels.

**Figure 2:**
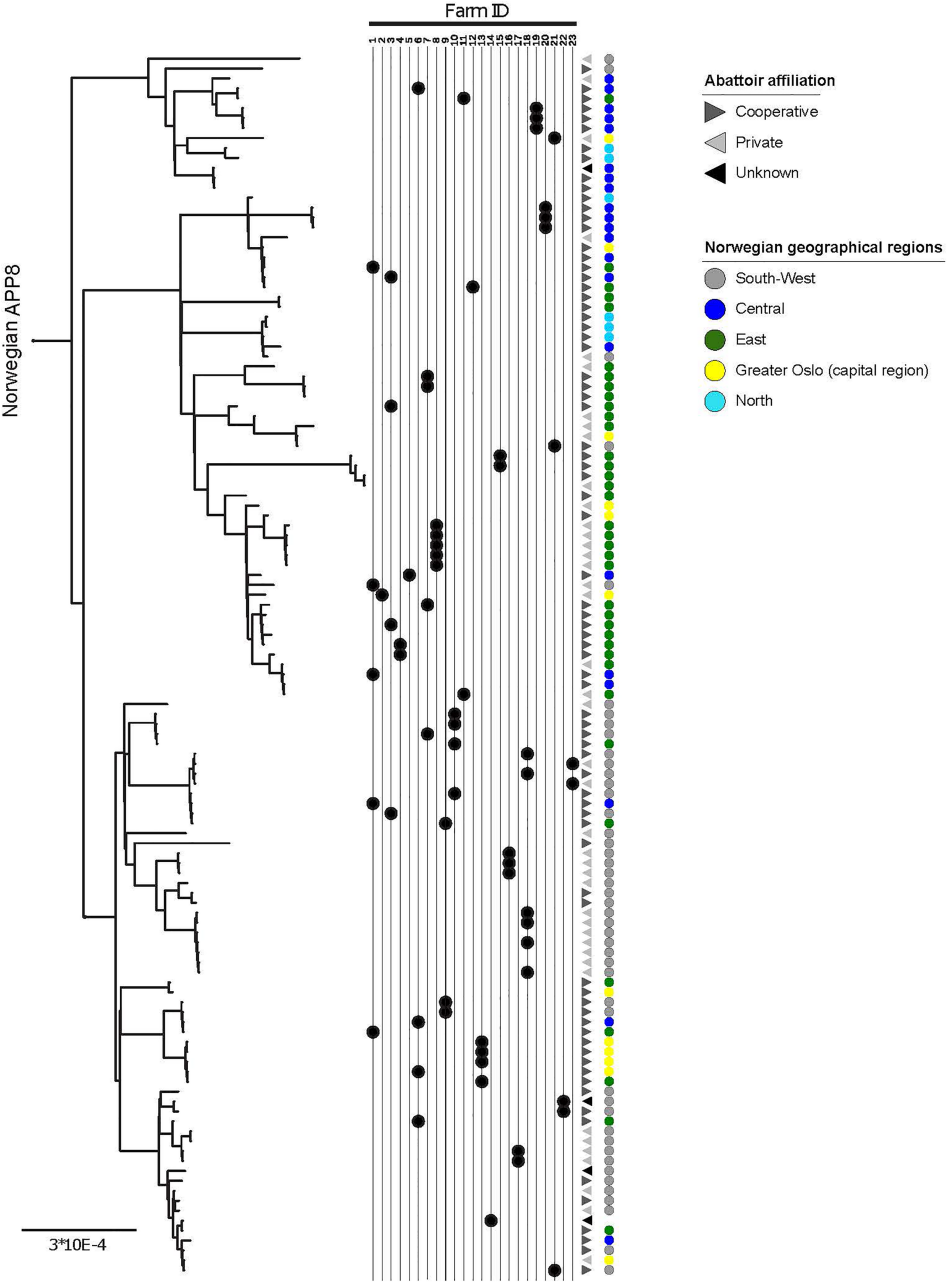
Within-herd variability, geographic origin, and abattoir affiliation of APP8. More than one isolate was sampled from each of 23 herds, indicated by the Farm ID, the black dots point to the location of the isolate in the phylogeny. Geographic origin across five major regions of Norway is indicated by colored circles (legend in figure). Abattoir affiliation to the two main slaughterhouse groups in Norway (Cooperative and Private) and a third Unknown group are indicated with greyscale triangles (legend in figure). The scale indicates substitutions per site.

Three distinguishable genetic clades were observed among the Norwegian isolates in the phylogeny and named Norway I, Norway II, and Norway III (Figure 1B). Median and maximal pairwise SNP distances within the Norwegian population were 112 and 153SNPs respectively, within a core genome of 1.67Mbp (71.6% of the reference chromosome), indicating a small within-population variation. Clonal isolates (<3SNPs) were in some cases isolated from pigs in the same herd. In other instances, isolates from pigs in the same herd sampled at the same point in time displayed a much greater variation (Figure 2).

#### Year of Sampling and Epidemiology of Isolates

The Norwegian isolates were sampled between 2004 and 2020, with a higher sampling frequency in 2013 and between 2017 and 2019 (Supplementary Figure 1). Samples from different years were scattered across the phylogeny, with few SNPs between isolates sampled up to 13years apart, suggesting that APP8 is diversifying at a slow rate in the population.

The clades within the Norwegian phylogeny showed a geographical pattern (Figure 2; Supplementary Figure 1). The χ^2^ of the distribution of isolates between three identified genetic clades and the three combined region categories (Table 4) was 72.3 (*p*<0.001). Accounting for herd as a random effect did not influence the results, hence the associations between phylogenetic clade and region was not random, meaning that isolates from the same geographic regions were more closely related than isolates from different regions. This finding supports geography as a factor of influence to the molecular evolution of *A. pleuropneumoniae*, and that different lineages of the bacterium spread within distinct geographic regions.

**Table 4:** Distribution of Norwegian isolates of APP8 (*n*=123) from three phylogenetic clades across three geographical regions in Norway (% of clade total).

Phylogenetic clade	Region 1	Region 2	Region 3	Unknown	Total
Norway I	2 (14.3)	2 (14.3)	10 (71.4)	0	14
Norway II	34 (66.7)	3 (5.9)	14 (27.4)	0	51
Norway III	13 (22.4)	41 (70.7)	3 (5.2)	1 (1.7)	58

Region 1=East and Greater Oslo, Region 2=South-West, and Region 3=North and Central Norway.

The primary repository isolates were mainly (i.e., 101 of 123) sampled from commercial herds. The distribution of herds in the different herd categories is presented in Table 2.

There was a visual clustering of isolates within the same abattoir system categories to phylogenetic clade (Figure 2), however the statistical analysis did not support this (χ^2^ 4.1, *p*=0.4).

### Within-Host Variation

A SNP distance ≤2 (median value between 0 and 1) was found between the isolates from the same pig, indicating that they belonged to the same clone. Further statistics on these isolates is shown in Table 3.

### Comparative Genome Analyses

#### Phylogenies

The APP8 isolates from UK and Norway were separated by approximately 1,500SNPs into two distinct phylogenetic branches (Figure 1A), where three Danish isolates were found scattered across the UK branch, while the remaining 19 Danish isolates clustered in two monophyletic groups in the Norwegian branch (Figure 1B).

#### Coalescence Analyses

The BEAST analysis performed on the Norwegian branch indicated that the APP8 population separated several centuries ago (Figure 1C). The last common ancestor with a Danish isolate, based on strict mutation rates and a coalescent Bayesian skyline tree prior, can be dated back to at least 200years (95% HDP: 1343–1837). From this analysis, we extrapolated that the Norwegian and UK isolates shared a common ancestor much further back in time, and that no evidence of later introductions can be found in our data. In contrast, our data support recent *A. pleuropneumoniae* transmissions between the UK and Danish pig populations which are reflected in the molecular relationships of APP8 isolated during the last decades.

#### AMR Genes

**Figure 3:**
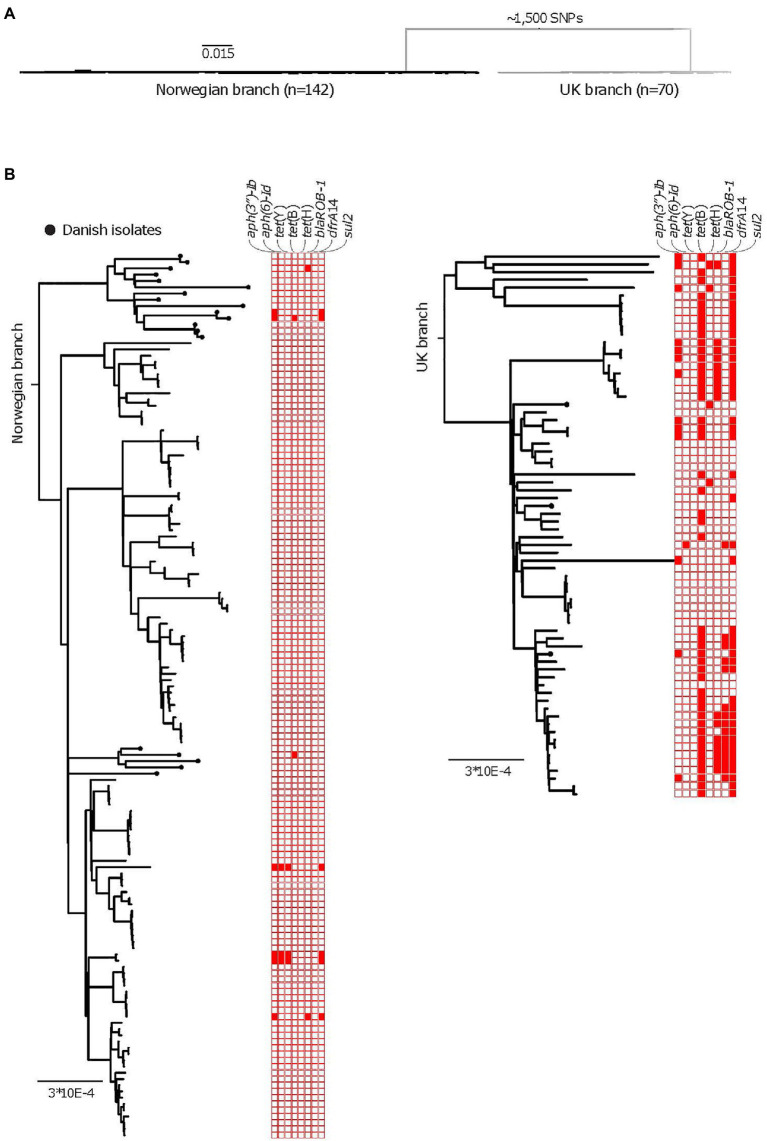
AMR among the APP8 populations collected over 13years. **(A)** A midpoint rooted phylogeny of isolates of APP8, after removal of recombinant regions, illustrating the two distinct branches of the APP8 collection (the Norwegian branch and the UK branch). The Norwegian branch contains 19 Danish and 123 Norwegian isolates (*n*=142), whereas the UK branch contains three Danish and 67 UK isolates (*n*=70) with ~1,500 SNPs separating the branches. **(B)** Rooted phylogenies of the Norwegian branch (left) and the UK branch (right), including AMR gene plot for streptomycin resistance genes *aph(3'')-Ib* and *aph(6)*, tetracycline resistance genes *tet*(Y), *tet*(B), and *tet*(H), beta-lactam resistance gene *bla*_ROB-1_, trimethoprim resistance gene *dfrA14* and sulfonamide resistance gene *sul2*. Presence of a resistance gene is indicated by a red colored box, while an empty box indicates absence of resistance gene. The scale indicates substitutions per site.

The following AMR genes were identified across the entire APP8 collection: *aph(3″)-Ib*, *aph(6)-Id* (streptomycin resistance genes, also called *strA* and *strB*, respectively), *tet*(Y), *tet*(H), *tet*(B; tetracycline resistance), *dfrA14* (trimethoprim resistance), *bla*_ROB-1_ (beta-lactam resistance), and *sul2* (sulfonamide resistance). We found statistical differences in occurrence of resistance genes in the three national populations, with the AMR genes most abundant among the UK APP8 isolates (Figure 3B; Table 5). The sulfonamide resistance gene, *sul2*, was the most common AMR gene occurring in the entire collection, being present in 3.3% of the Norwegian and 66% of the UK isolates. The resistance profiles of a large collection of UK isolates, including the ones in this study, have been described previously (Bossé et al., 2017). In the UK APP8 branch, the AMR genes were more prevalent in some subclusters, suggestive of a clonal expansion and/or local dissemination by acquisition of mobile genetic elements. The three Danish isolates within the UK branch also resembled the UK isolates in terms of AMR genes. The remaining 19 Danish isolates were nested within the Norwegian branch and had similar low prevalence of AMR genes.

**Table 5:** AMR genes identified in isolates of APP8 from Norway (*n*=123), Denmark (*n*=22), and the United Kingdom (*n*=67).

Country of origin	*aph(3'')-Ib*/*strA*	*aph(6)-Id*/*strB*	*tet*(Y)	*tet*(H)	*tet*(B)	*dfrA14*	*bla*_ROB-1_	*sul2*	Total
Norway	3.3% (4)	2.4% (3)	2.4% (3)	-	-	-	0.8% (1)	3.3% (4)	3.3% (4)
Denmark	9.1% (2)	-	-	4.5% (1)	13.6% (3)	4.5% (1)	4.5% (1)	13.6% (3)	22.7% (5)
United Kingdom	19.4% (13)	1.5% (1)	-	4.5% (3)	67.2% (45)	22.4% (15)	23.9% (16)	67.2% (45)	74.6% (50)

*aph(3'')-Ib* and *aph(6)-Id*=streptomycin resistance genes (also called *strA* and *strB*, respectively); *tet*(Y), *tet*(H), and *tet*(B)=tetracycline resistance genes; *dfrA14*=trimethoprim resistance gene; *bla*_ROB-1_=beta-lactam resistance gene; *sul2*=sulfonamide resistance gene. The number of isolates harboring the gene is given in parentheses. Genes that were not present are indicated by “-”.

Within the isolates from the Norwegian population, AMR genes were found in only 3.3% (*n*=4) of the isolates. The four isolates in question did not cluster together, pointing to independent acquisition of the AMR genes. The four isolates originated from the same abattoir system, in three different regions, namely Southwest, East and Greater Oslo, and were isolated over a span of 13years. A combination of the genes *aph(3″)-Ib*, *aph(6)-Id*, *tet*(Y), and *sul2* was found in three of these. To our knowledge, this is the first time *tet*(Y) has been found within the *Pasteurellaceae* family.

## Discussion

In this study we characterized the genetic variability in terms of SNPs, to infer molecular relationships, and distribution of AMR genes in a collection of APP8 isolates. We found that both internationally and within Norway, geographic origin is associated with molecular relationships, as we observe distinct genetic clustering between countries and within major geographical regions in Norway. By applying coalescence analyses, we estimated that the Norwegian isolates separated from UK and Danish isolates several centuries ago. This distinct genetic separation may be due to several historical aspects. Live animal contact between the UK and Norway has not been documented in recent times, reflected by the distinct separation of the APP8 phylogenies of these countries into two branches. Our data show no signs of recent contact. The separation of the UK and Norwegian branches seems to date back to the Middle Ages, during which Vikings from Norway traveled by boat to the British Isles and were likely to bring livestock out or back home. The clustering of Danish isolates within the UK and Norwegian branches are indicative of multiple introductions, also estimated to have occurred at times where live animal exchange was more likely than today. Evidence of contact between the Norwegian and Danish populations is indicated in the phylogeny to have last occurred around 200years ago. Norway was under Danish rule in the period between 1537 and 1814. Livestock exchange was more likely to have occurred during this period, although no such records have been tracked in the writing of this manuscript. Breeding animals from UK were introduced in Denmark in the 1980s and is probably the reason for the detection of three Danish APP8 isolates among the UK isolates (personal communication, Øystein Angen, senior researcher at SSI, Copenhagen).

By applying geographic and population structure data to our phylogeny, we observed a significant genetic clustering of our isolates. Closely related isolates were identified within geographic regions, supporting geography as a factor of influence to the molecular evolution of *A. pleuropneumoniae*. Surprisingly, there was no clustering of the abattoir categories in the phylogeny, supporting direct contact as the main source of transmission, since livestock trade usually is restricted within these systems. Strains of *A. pleuropneumoniae* are believed to persist within the breeding herds, harbored in the tonsils and chronic lesions of adult sows (Fablet et al., 2011). Since most of these herds are self-supplying, no direct contact with animals from other herds takes place. One nucleus breeding herd can supply pigs (mainly gilts) directly to several multiplier breeding herds, which in turn can supply pigs to many commercial herds, usually located in the same part of the country. This enables transmission of clonal isolates, allowing their persistence within the system and geographic region. Additionally, individual commercial fattening pig herds can purchase livestock from multiple sow herds, in which case we would expect the pigs to be carrying genetically different strains. This is a reasonable explanation for the observed range in genetic within-herd variability but was not investigated further.

At the individual host level, we observed almost no genetic variation. This is likely due to inherent biologic characteristics of the bacterium and the host. It has been shown that pigs can carry a variety of *A. pleuropneumoniae* isolates in their tonsils (Vigre et al., 2002). The isolates can differ in serovar and potential for invasive infection because of virulence factors that enable them to colonize the lower respiratory tract. Diseased and infectious pigs can transmit the clinical isolate through aerosols by coughing, which is a common clinical sign, and in their saliva and nasal secretions by nose-to-nose contact to susceptible pigs (Gottschalk and Broes, 2019). Outbreaks of porcine pleuropneumonia within affected herds are common, though it is not well described in literature if these occur due to newly introduced virulent strains of *A. pleuropneumoniae* and/or by descent to the lower respiratory tract of strains already resident in the tonsils. However, it has been suggested that in endemically infected herds where *A. pleuropneumoniae* is harbored asymptomatically in the tonsils, environmental triggers (such as stress or co-infection with other respiratory pathogens) play a larger role in precipitating disease outbreaks than transmission of a newly introduced virulent strain (Klinkenberg et al., 2014). During an outbreak of disease, the infectious pressure of more virulent isolates will increase, and if descending bacteria from the tonsils are also involved, a variation in the genomes of bacteria isolated from the lungs of diseased pigs is expected. When diagnosing a case of porcine pleuropneumonia in a herd, due to practical and financial considerations, it is not uncommon that only a single sample per pig or per herd is submitted for in-depth diagnostics including serotyping and AMR testing. Our results support that porcine pleuropneumonia in a pig is caused by a monoclonal infection, indicating that a single sample per pig will be sufficient for a diagnostic purpose. Still, different isolates of APP8 were isolated within a herd, meaning that the mechanisms of disease in a herd was not solely tied to the spread of a single virulent clone. As virulence can vary both between different serovars of *A. pleuropneumoniae* and between isolates of the same serovar (Sassu et al., 2017), there is a possibility that our findings do not apply across all serovars. Our results must still be considered valuable when establishing relevant control strategies against *A. pleuropneumoniae*.

The pangenome of six Brazilian APP8 isolates was recently reported (Prado et al., 2020) and showed that the gene repertoire is well conserved in relation to the available genomes of other serovars, though the presence of serovar-specific patterns of AMR within *A. pleuropneumoniae* is debatable (Asawa et al., 1995; Lee et al., 2015; Kim et al., 2016). Due to a low prevalence of clinical pleuropneumonia caused by APP8 globally, most studies on AMR have been performed on other serovars. AMR genes have many times been found to be linked to, and accumulate in, mobile genetic elements, acting as vehicles for horizontal transmission in many bacterial species. *A. pleuropneumoniae* is no exception (Bossé et al., 2015, 2016b), and many isolates in our study harbor multiple resistance genes that could be tied to such mobile elements. Mobile genetic elements, like plasmids and Integrative and Conjugative Elements (ICE), are not generally tied to specific serovars, though integration of ICE into the chromosome does favor vertical in addition to horizontal transmission, perhaps explaining why some elements have only been found within specific serovars so far. The *tet*(B) gene has previously been shown to be part of a Tn*10* insertion in an integrative conjugative element, ICE*Apl*1 (Bossé et al., 2016b), in the MIDG2331 genome (Bossé et al., 2016a) and in the genomes of a further 21 UK clinical APP8 isolates (Bossé et al., 2017). This gene was also associated with a Tn*7* insertion in the chromosome in 15 (one serovar 7 and the rest APP8), and with possible plasmid sequences in 14 (one each of serovars 2, 6, 7, and 12, and the rest APP8) further isolates (Bossé et al., 2017). The other AMR genes, i.e., *tet*(H), *sul2*, *dfrA14*, *bla*_ROB-1_, *aph(3″)-Ib*, and *aph*(*6*; previously referred to as *strA* and *strB*), were all associated with potential plasmids, in a variety of serovars, as indicated by the sequences flanking the AMR genes on the associated contigs (Bossé et al., 2017). The presence of other AMR bacteria in the host population is likely a risk factor for acquisition of these genetic elements. National strategies for handling emergence of multidrug-resistant bacteria in livestock in general could therefore be of relevance to prevent development of AMR in clinically important pathogens like *A. pleuropneumoniae*. It is of high interest to the Norwegian pig production sector to avoid introducing multi-resistant bacteria through contact with other pig populations. Our results conclude that important differences are present also within a serovar, and factors other than the intrinsic properties of serovars contribute to the AMR biodiversity.

The observed differences in AMR gene distribution can also be attributed to the deviating treatment practices in the respective countries. Substantial differences in antimicrobial use exist in pig production in Norway, the UK, and Denmark (Veterinary Medicines Direcorate, 2019; DANMAP 2019, 2020; NORM/NORM-VET 2019, 2020). Sulfonamides were the most used subgroup of antimicrobial drugs for treatment of production animals in Denmark in the 1980s, bypassed by tetracyclines in the 1990s. Resistance to sulfonamides and tetracyclines was found in Danish *A. pleuropneumoniae* isolates already in 1995 (DANMAP 1996, 1997). Around this time, the Danish government implemented measures to significantly reduce the use of antimicrobial drugs in food production. AMR in *A. pleuropneumoniae* has not been systematically investigated in Norway. According to current Norwegian therapeutic guidelines, benzylpenicillin-procaine is the drug of choice for treating porcine pleuropneumonia (Norwegian Medicines Agency, 2012). In a recent field study of acute outbreaks of porcine pleuropneumonia in Norway (Cohen et al., 2020), treatments were found to be in line with these recommendations. In comparison, tilmicosin and tulathromycin have been commonly used in Denmark against acute pleuropneumonia partly due to the convenience of peroral administration (DANMAP 2019, 2020), which is not common practice in Norway (European Medicines Agency, 2018). In the UK, a systematic decrease in general antimicrobial drug use for pigs has been observed since 2015 across most relevant drug classes. However, tetracyclines remain the most used drug class (UK-VARSS, 2020), possibly contributing to the continued selection of tetracycline resistance in *A. pleuropneumoniae*.

We studied genetic variability in three different levels, i.e., within-host, within-population and between populations. The generalizability for the UK and Danish isolates was somewhat hard to ascertain due to limited access to metadata. Isolates were collected from all levels of the production system, and from all the major pig producing regions in Norway. Overall, we observed a low within-population variability of APP8 in Norway and a persistence of genetic lineages over time. Time is only one factor to influence variability, however it is worth noting that our data consists mainly of isolates collected after 2004 and are not equally represented in time. This concentrated sampling contributes to the reported uncertainty around the estimates of population divergence between countries. Since sampling from the whole population is not feasible, an adequate representation of the population over time is necessary for good estimates of divergence. However, we believe that the isolates in our material have been subject to minimal selection bias due to being passively collected through routine diagnostics. A baseline phylogeny of *A. pleuropneumoniae*, as provided by this study, will likely be of great value to future surveillance and control of this pathogen, both because it increases our understanding of the effects of restricted animal movement, and as it enables the discovery of introductions of new genetic lineages. To unveil the clinical relevance of these genetic characteristics, future studies on pathogenicity within genetic lineages are necessary.

## Conclusion

In this study we utilized genomic data from populations of APP8 to elucidate the population dynamics of the pigs. With modern sequencing techniques and genomic analyses, we were able to study genetic variability, both within and between populations, and to identify evolutionary patterns and relationships. Isolates sampled within-host were nearly identical, and there was little genetic variability between isolates from pigs in a herd during an outbreak, supporting that one sample per animal and only a few samples per herd should suffice for diagnostic sampling. The occurrence of AMR genes in Norwegian isolates is low, and there is a substantial difference in the occurrence of AMR of APP8 in Norway and the UK. By applying relevant metainformation about the source of the strains, we increased our understanding of their correlation to genetic traits such as AMR. Likely a result of the closed pig population strategies, there is no evidence of recent transmission of *A. pleuropneumoniae* into Norway from Denmark and the UK, and the last common ancestor dates more than 200years back. Our results indicate that the genetic variability found within and among the APP8 populations is influenced not only by inherent biologic characteristics that affect the transmission of the bacteria, but also heavily influenced by social and political strategies and regulations that affect the host population dynamics. A baseline phylogeny of *A. pleuropneumoniae* as provided by this study, will likely be of great value to future surveillance and control of this pathogen, partly because it increases our understanding of the effects of restricted animal movement nationally and internationally and enables the discovery of introductions of new genetic lineages.

## Data Availability Statement

The datasets presented in this study can be found in online repositories. The names of the repository/repositories and accession number(s) can be found at: https://www.ebi.ac.uk/ena/browser/view/PRJEB47034, ENA, PRJEB47034.

## Author Contributions

ØA, JB, CG, CK, BR, SG, TK, MS, PL, YL, and LC conceptualized and designed the study. ØA, YL, and LC handled the bacterial samples. MS and ØA performed the bioinformatic analyses. MS, ØA, CK, JB, and LC analyzed the data and interpreted the results. JB and LC wrote the main body of the manuscript. All authors contributed to the article and approved the submitted version.

## Funding

We are grateful for the funding provided by the Agricultural Agreement Research Fund and the Foundation for Research Levy on Agricultural Products, grant number NFR-267422, and the UK Biotechnology and Biological Sciences Research Council (BB/S002103/1, BB/S005897/1, and BB/G018553/1). We extend our gratitude to our collaborators who also funded the research: Animalia, Nortura and KLF. The Norwegian Veterinary Institute, Statens Serum Institute and Imperial College covered all costs related to sample handling, storing, and shipping.

## Conflict of Interest

The authors declare that the research was conducted in the absence of any commercial or financial relationships that could be construed as a potential conflict of interest.

## Publisher’s Note

All claims expressed in this article are solely those of the authors and do not necessarily represent those of their affiliated organizations, or those of the publisher, the editors and the reviewers. Any product that may be evaluated in this article, or claim that may be made by its manufacturer, is not guaranteed or endorsed by the publisher.

## Footnotes

^1^https://github.com/ssi-dk/bifrost (Accessed April 09, 2021).

^2^https://itol.embl.de (Accessed April 09, 2021).

^3^https://microreact.org (Accessed April 09, 2021).

^4^https://github.com/tseemann/abricate (Accessed April 09, 2021).

^5^https://www.phylo.org (Accessed April 23, 2021).
