# Supplementary material for: Comparative Genome Sequence Analysis of Actinobacillus pleuropneumoniae Serovar 8 Isolates From Norway, Denmark, and the United Kingdom Indicates Distinct Phylogenetic Lineages and Differences in Distribution of Antimicrobial Resistance Genes
Source: Front Microbiol. 2021 Sep 10;12:729637. doi: 10.3389/fmicb.2021.729637 (PMC8461171; doi:10.3389/fmicb.2021.729637)
Supplement: Supplementary file 2 [file Image_1.PDF]

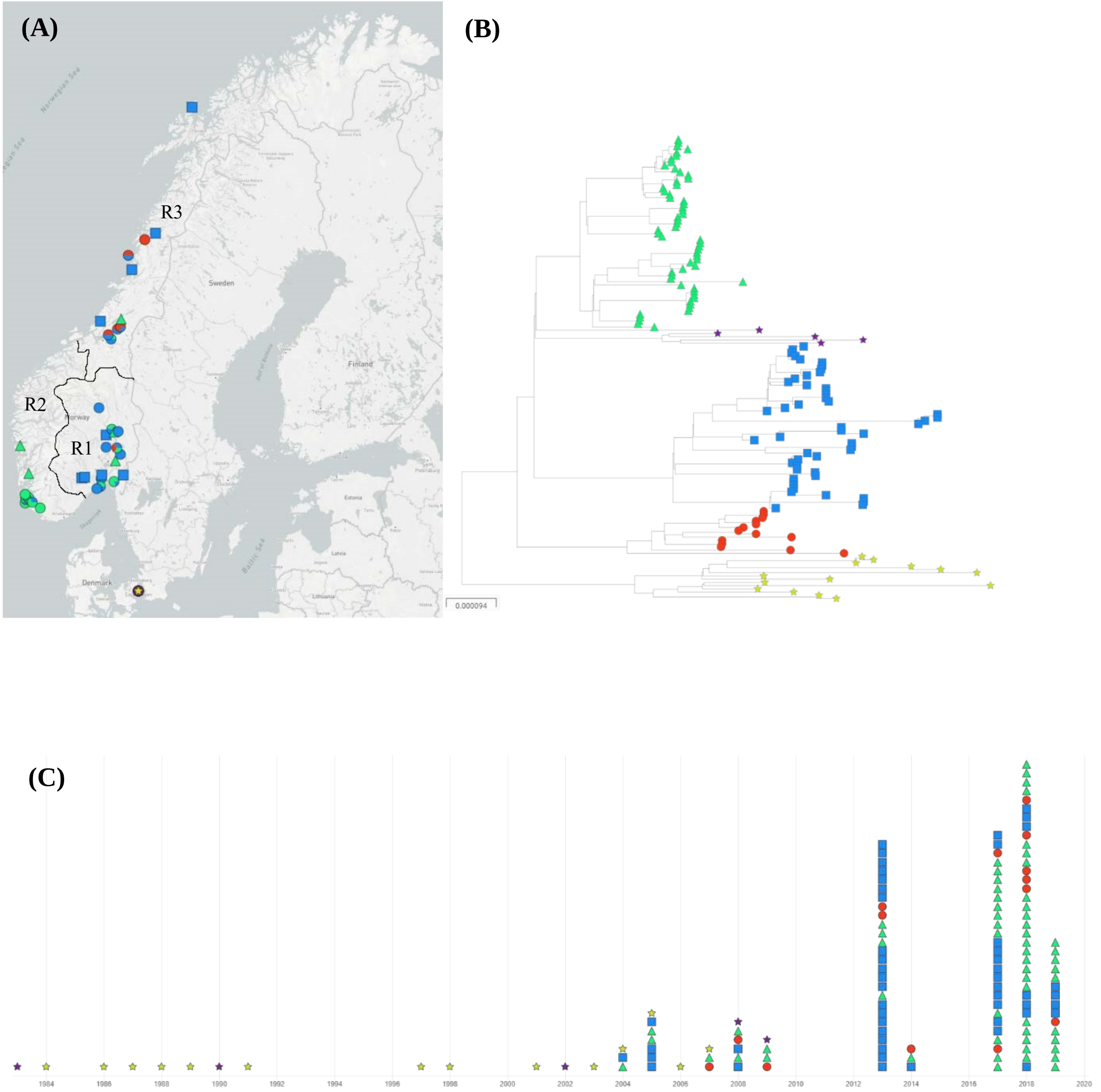

Supplementary Figure 1 Geographical **(A)**, phylogenetic **(B)** and temporal **(C)** distribution of *Actinobacillus pleuropneumoniae* serovar 8 isolates from Norway and Denmark. Geographical regions of Norway are indicated by labels, R1 = East and Greater Oslo, R2 = South-West, R3 = North and Central. Phylogenetic clades are indicated by shape and color of marker, Norway I = red circle, Norway II = blue square, Norway III = green triangle, Denmark I = yellow star, Denmark II = purple star, grouping according to Figure 1. Each Norwegian isolate was given the coordinates for the capital of the municipality that the farm was located in out of practical and privacy considerations. The Danish isolates are all given the same coordinates as the farm of origin is not known. The timeline (bottom) indicates the year of isolation.
